# Untargeted metabolomics: an emerging approach to determine the composition of herbal products

**DOI:** 10.5936/csbj.201301007

**Published:** 2013-02-28

**Authors:** Mauro Commisso, Pamela Strazzer, Ketti Toffali, Matteo Stocchero, Flavia Guzzo

**Affiliations:** a University of Verona, Department of Biotechnology, Strada le Grazie 15, Cà Vignal 1, 37134 Verona, Italy; b S-IN Soluzioni Informatiche, Via Salvemini 9, 36100 Vicenza, Italy

**Keywords:** LC-MS, herbal products, herbal intoxications, natural remedies, *Daucus carota*, Multivariate Analysis

## Abstract

Natural remedies, such as those based on traditional Chinese medicines, have become more popular also in western countries over the last 10 years. The composition of these herbal products is largely unknown and difficult to determine. Moreover, since plants respond to their environment changing the metabolome, the composition of plant material can vary depending on the plant growth conditions.

However, there is a growing need of a deeper knowledge on such natural remedies also in view of the growing number of reports of toxicity following the consumption of herbal supplements. Untargeted metabolomics is a useful approach for the simultaneous analysis of many compounds in herbal products. In particular, liquid chromatography/mass spectrometry (LC-MS) can determine presence, amount and sometime structures of plant metabolites in complex herbal mixtures, with significant advantages over techniques such as nuclear magnetic resonance (NMR) spectroscopy and gas chromatography/mass spectrometry (GC-MS).

## 1) Introduction

Plants have provided humans with medicines since the dawn of civilization, and up to 80% of the population in many Asian and African countries still depends on traditional herbal medicines for primary health care [1]. In western countries, herbal medicines were supplanted by modern drugs with defined pharmaceutical ingredients, but their popularity is increasing once again, particularly for the alleviation of mild disease symptoms. Approximately one third of the UK adult population now uses herbal products [2] and the market for herbal medicines in the USA was $US 5 billion in 2010 [3]. Herbal medicines raise a number of safety issues such as the identification of plant materials in herbal products, the active principles, the method of preparation, dosing regimens, the potential to interact with other herbal remedies and conventional drugs, and assurances that herbal products are genuine and do not contain toxins or contaminants. The lack of clear safety guidelines for herbal medicines has resulted in many reports of toxic effects, such as the 13 cases of hepatic toxicity leading to the withdrawal of a green tea product used for weight loss, which was distributed by Exolise (Arkopharma, Carros, France) [2]. Green tea (prepared from *Camellia sinensis* leaves) is a popular herbal product that contains polyphenols such as catechins, which are thought to promote health by reducing the risk of cardiovascular disease, diabetes and obesity [4]. The cluster of hepatic toxicity cases mentioned above was probably caused by high levels of epigallocatechin gallate (EGCG) [5]. Although low doses of EGCG appear to be safe and even beneficial, it is impossible to control the dosage of herbal products as effectively as formulated pharmaceuticals [6].

Other plants often used in herbal preparations (e.g. *Heliotropium* spp., *Senecio* spp., *Symphytum* spp., *Crotalaria* spp. and *Borago officinalis*) accumulate high levels of pyrrolizidine alkaloids, which can induce veno-occlusive disease (VOD) associated with acute liver failure or liver fibrosis and cirrhosis [5]. *Valeriana officinalis* is a popular herbal product used to alleviate insomnia but extracts are known to induce severe hepatitis, supported by *in vitro* studies that demonstrate the inhibition of cytochrome P450 isoforms CYP3A4, CYP2D6 and CYP2C19 [7].

The regulation of herbal products marketing has been attempted through initiatives such as the Traditional Herbal Medicinal Products Directive (THMPD) which came into force throughout the European Union (EU) in April 2011 [2]. Under this directive, a plant product is eligible for registration as a traditional herbal medicine only if it has been used as treatment for specific minor ailments for at least 30 years, including a minimum of 15 years in Europe. The efficacy and safety of such plant products is accepted only if a long history of use is demonstrated. However, no clinical trials are required, which sets traditional herbal medicines aside from newer herbal products and defined pharmaceutical ingredients.

In the USA, herbal products are regulated as dietary supplements, and no claims that they prevent diseases are allowed, so FDA registration and chemical analysis are not required. However, if specific health claims are made, then the products must be registered as drugs and must undergo the rigorous FDA approval process [2].

Beside the existing national or over-national laws, the effective safety and regulation of herbal products do require methods that can provide convincing evidence of clinical efficacy (in some cases through clinical trials) and powerful analytical tools that can determine the composition of herbal products and identify the active principles. Metabolomics appears ideal for this purpose. According to the Metabolomics Society home page, metabolomics can be defined as the “comprehensive characterization of the small molecule metabolites in a biological system” (http://www.metabolomicssociety.org/). In turn, such small metabolites represent the outcome of gene expression and define the biochemical phenotype of a cell, tissue, organ and organism. The small metabolites include the intermediates and end products of metabolism, and they compass both primary metabolites (e.g. sugars, amino acids, fatty acids and organic acids) and secondary metabolites (e.g. phenylpropanoids and alkaloids). Metabolomics can also characterize the dynamic metabolome, reflecting changes in the abundance of small molecules during development and in response to external stresses.

The most popular analytical methods in metabolomics are those based on nuclear magnetic resonance (NMR) spectroscopy and mass spectrometry (MS). NMR allows the rapid, high-throughput and automated analysis of crude extracts, and the quantitative detection of many different groups of metabolites [8, 9], providing also structural information including stereochemical details [10]. However, NMR is less sensitive than MS-based approaches [9] and NMR data have been metaphorically compared to ‘the tip of the iceberg’, with LC-MS providing details of the much larger, submerged portion [11].

Between these extremes, GC-MS is particularly suitable for the detection of thermally stable volatile compounds (or compounds with volatile derivatives). LC-MS is more sensitive than GC-MS and it allows the analysis of thermally labile non-volatile compounds [12]. The molecules that can be detected by LC-MS range from polar sugars and non-aromatic organic acids [13] through to various lipids [14], as discussed in recent reviews [9, 12, 15]. Here we focus on the use of LC-MS for untargeted metabolomics, allowing the composition of complex plant tissues and their products to be unraveled.

## 2) LC-MS untargeted metabolomics allows the comprehensive analysis of complex plant tissues

**Figure 1:**
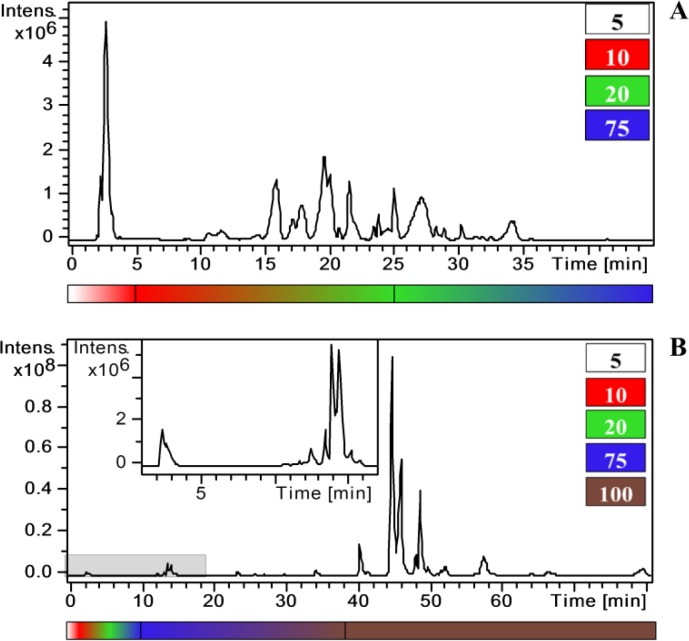
Two examples of LC-MS analysis, showing chromatograms representing two different matrices obtained using different ion sources. The x-axis shows the time in minutes and the y-axis shows the relative signal intensity. The underlying bars represent the chromatographic elution patterns which start at 5% (white) and increase to 75% (blue) or 100% (brown) of acetonitrile. In the chromatograms, polar compounds elute first, followed semi-polar and non-polar compounds. **A:** LC-ESI-MS/MS chromatogram of a grape berry methanolic extract. **B:** LC-APCI-MS/MS chromatogram of an *Arabidopsis thaliana* methanolic extract.

The ability to analyze various kinds of metabolites by LC-MS depends strongly on the ionization source and the chromatographic method used for analyte fractionation and elution. Two examples are provided in Fig. 1. The first is a grape berry methanolic extract, analyzed using an electrospray ionization (ESI) source and reverse phase chromatography with gradient elution rising to 75% acetonitrile, allowing the detection of sugars, aromatic and aliphatic organic acids, anthocyanins, flavonoids and stilbenes (Fig. 1A and Table 1). The second is an *Arabidopsis thaliana* methanolic extract, analyzed using an atmospheric pressure chemical ionization (APCI) source and reverse phase chromatography with prolonged equilibration in 100% acetonitrile, allowing the additional detection of polar lipids such as monogalactosyl diacylglycerol and digalactosyl diacylglycerol (Fig. 1B). Therefore, among the available approaches, LC-MS-based untargeted metabolomics combines high sensitivity and an untargeted approach to provide an ideal procedure for the analysis of a wide range of non-volatile metabolites.

**Table 1:** Metabolites detected in grape extracts, using LC-MS in negative or positive ion modes.

Metabolite class	Ionization mode	Metabolites
Sugars	Negative	Sucrose, hexoses, small oligomers
Aminoacids	Positive	-
Flavan-3-ols	Negative and positive	Catechin, epicatechin, procyanidins
Anthocyanins	Negative and positive	Non-acylated anthocyanins, anthocyanins acylated with acetic, coumaric and caffeic acid
Stilbenes	Negative and positive	Resveratrol, resveratrol oligomers, viniferins
Non-aromatic organic acid	Negative	Tartaric, citric, malic acid
Hydroxycinnamic acid derivatives	Negative	Caffeic, coumaric and ferulic acd derivatives, hydroxytirosol derivatives
Hydroxybenzoic acid derivatives	Negative	Dihydroxybenzoic acid, vanillic acid, gallic acid derivatives

### 2.1) Workflow of a typical LC-MS untargeted metabolomics experiment

**Figure 2:**
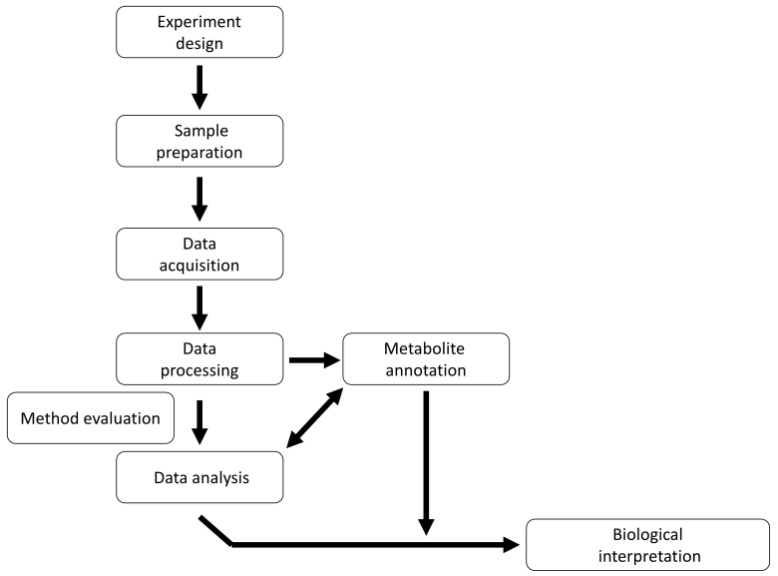
Typical untargeted metabolomics workflow.

A flow chart of a typical LC-MS untargeted metabolomics experiment is shown in Fig. 2. Crucial points include the experimental design, the extraction protocol, data acquisition, processing and analysis, and metabolite identification to allow biological interpretation. The experimental design, extraction and data acquisition methods depend on the aim of the investigation, on the material used for analysis and the available instrumentation [16]. Several recent studies have considered the challenge of data processing in untargeted metabolomics, which has benefited enormously from the development of automated procedures [15, 17]. Recent data-processing tools such as MetAlign (http://www.metalign.wur.nl), MZmine (http://mzmine.sourceforge.net/) and XCMS (http://metlin.scripps.edu/download/) [18] are designed to extract relevant information automatically from batches of crude chromatographic data, allowing the rapid processing of thousands of data points, which transforms the concept of untargeted metabolomics into practical reality. The principal challenges these tools must overcome include background subtraction, signal recognition, signal quantification and signal alignment, so that the output of a batch of LC-MS chromatograms is usually a table listing the amount of each signal in all samples.

### 2.2) Data analysis in untargeted metabolomics

After data processing, data analysis can follow one of two major routes [15]. If the aim is to profile a small number of specific metabolites, usually the more abundant ones (targeted metabolomics), quantitative methods are used to analyze the targeted variables one by one and statistical significance can be tested using univariate methods or other classical statistical approaches. In contrast, if the aim is to provide a holistic picture of the system under investigation (untargeted metabolomics), a large number of known and unknown metabolites are quantified and all the obtained variables are considered simultaneously, making univariate and other classical statistical methods unfeasible. Indeed, to take into account the correlation structures between the measured variables, multivariate statistical methods must be applied. Both targeted and untargeted metabolomics reveal the expected behavior of known metabolites, but only untargeted metabolomics also allows the detection of synergic effects between variables which cannot be observed at an individual level. Untargeted metabolomics is usually a hypothesis-free approach. The most widely-used multivariate techniques are those based on projection. Projection methods combine the measured variables into so-called latent variables that can solve the problem under investigation. For exploratory data analysis Principal Component Analysis (PCA) can be successfully applied. PCA is an unsupervised technique that can summarize the information in an experimental data set using a small number of orthogonal latent variables obtained by searching the direction of maximum variance in the data set. However, PCA does not always extract hidden information that explains system behavior because this may not correspond to the information summarized in the latent variables. Supervised techniques may be preferred for these cases. Indeed, training sets comprising samples with well-known properties can be used to drive the projection in directions that produce latent variables capable of solving the problem of interest. Latent variable regression methods such as Bidirectional-Orthogonal Projection to Latent Structures (O2PLS) [19] and their formulation for Discriminant Analysis have been used successfully to solve regression or classification problems. In many cases, the latent variables produced by projection can be interpreted in terms of single measured variables, and simple models can be built using a subset of the original data. In particular, when classification problems are considered single putative markers can often be highlighted by exploring the latent structure of the projective model.

### 2.3) Metabolite identification in untargeted metabolomics

The identification of metabolites can be a significant challenge in untargeted metabolomics, particularly because plants often transform secondary metabolites by glycosylation and the formation of esters to generate species-dependent metabolic profiles. These diverse molecules can be valuable in terms of their pharmacological properties but difficult to identify with accuracy. Several LC-MS metabolite feature databases have been assembled, such as MASSBANK (http://www.massbank.jp/index.html) [20], METLIN (http://metlin.scripps.edu/) [21] and MS2T (http://prime.psc.riken.jp/lcms/ms2tview/ms2tview.html) [22], but because of the sheer diversity of plant metabolites the coverage provided by databases remains inadequate. However, the accurate mass determination and fragmentation patterns obtained by tandem mass spectrometry (MS/MS), especially when combined with soft-ionization techniques such as ESI, can allow the elucidation of metabolite structures *de novo*. For example, high-performance liquid chromatography diode array detection (HPLC-DAD), electrospray ionization time-of-flight mass spectrometry (HPLC-ESI-TOF-MS) and electrospray ionization quadrupole ion trap mass spectrometry (HPLC-ESI-MS/MS) have been used for the isolation, identification and structural analysis of water-soluble phenolic and nonpolar diterpenoid constituents in danshen roots (*Salvia miltiorrhiza*) [23] and flavonoid compounds in extracts of *Dendrocalamopsis oldham* leaves [24]. In the latter study, the authors were able to determine the structures of 11 compounds, and thus identify four mono-C-glycosylflavones, three O,C-diglycosylflavones and three O-glycosylflavones, including three types of aglycone (luteolin, apigenin and tricin). In another example, ESI-MS/MS was combined with reversed-phase HPLC to identify proanthocyanidins in Saskatoon berries (*Amelanchier alnifolia*), confirming the presence of multimeric proanthocyanidin compounds ranging from dimers through to heptamers and even higher polymers [25].

## 3) LC-MS untargeted metabolomics can unravel environment-dependent plant cell composition

Plants are sessile organisms and must adapt to changes in their environment by modulating their developmental, physiological and biochemical responses. Plants benefit from the ability to synthesize a broad and diverse range of secondary metabolites, because such compounds allow them to adapt or survive when exposed to biotic and abiotic stress [26, 27]. The metabolic profile of plants can therefore change according to the environment, which means the active principles in herbal remedies can vary qualitatively and quantitatively depending on the growth conditions. From a quality control perspective, the active principles in herbal remedies need to be standardized, but this can be difficult in plants subjected to variable environments [28].

### 3.1) Responses of carrot cells to environmental conditions: an example of environment dependent cell composition unraveled by LC-MS untargeted metabolomics

**Figure 3:**
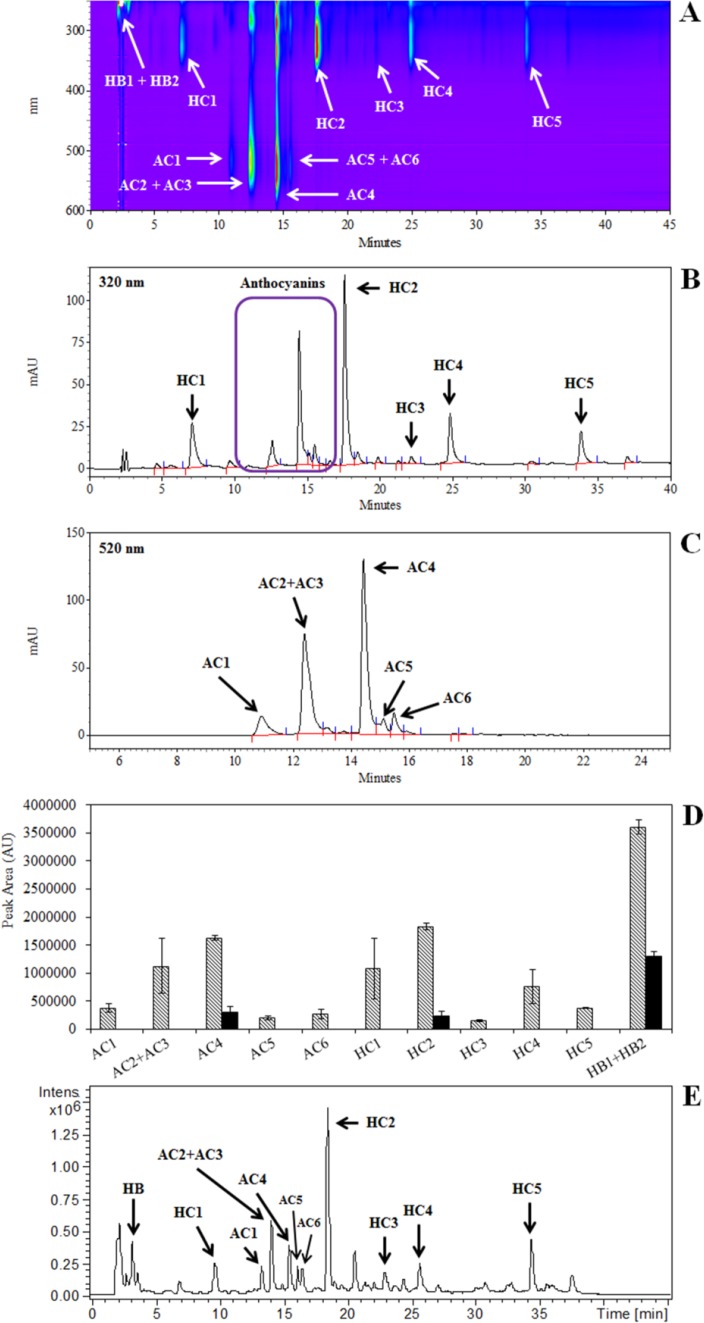
R3M carrot cell suspension extract analyzed by HPLC-DAD and HPLC-ESI-MS. **A:** Absorbance (245–600 nm) expressed in Arbitrary Units (mAU) using a scale is set from a minimum level of absorbance (-10 mAU) shown in purple, to a maximum (+100 mAU) shown in red. **B:** Chromatogram at 320 nm. **C:** Chromatogram at 520 nm. The peaks were characterized by LC-MS as follows. *AC1*: Cyanidin hexosyl pentosyl hexoside and cyanidin hexosyl pentoside. *AC2 + AC3*: Cyanidin caffeoyl hexosyl hexosyl hexoside and cyanidin caffeoyl hexosyl pentosyl hexoside. *AC4*: Cyanidin sinapoyl hexosyl pentosyl hexoside. *AC5*: Cyanidin feruloyl hexosyl pentosyl hexoside. *AC6*: Cyanidin coumaoryl hexosyl pentosyl hexoside. *HC1*: Caffeoyl quinic acid. *HC2*: Caffeoyl methyl quinic acid. *HC3*: Dicaffeoyl quinic acid. *HC4*: Caffeoyl methyl quinic acid and Caffeoyl dimethyl quinic acid. *HB1 and HB2*: Unknown compounds with absorbance spectra similar to hydroxybenzoic acids. **D:** Quantification of the 11 major peaks detected by LC-DAD. Black and white columns represent cells cultivated in constant darkness and under constant illumination, respectively. The values are the average of three independent biological replicates. **E:** LC-ESI-MS analysis of cells cultivated under constant illumination. Several peaks, which are not detected by LC-DAD, are also present in the chromatogram.

The challenging issue of determination of plant material composition requires adequate technologies in order to be properly faced. LC-MS-based untargeted metabolomics, as described in the flow chart of Fig. 2, is suitable to analyze the environmental variability of plant cell accumulated metabolites. A simple example (data unpublished) is the comparison of carrot suspension cells initially growing under standard conditions [29, 30], and then switched to constant light or darkness for one cell cycle (14 days). Methanolic extracts prepared from each of the cultures were analyzed by HPLC-DAD and HPLC-ESI-MS with the same methods described by Toffali *et al*. [31]. The resulting HPLC-DAD chromatogram comprised 11 peaks corresponding to various hydroxycinnamic and hydroxybenzoic acids, anthocyanins, flavonoids and two unidentified molecules (Fig. 3 A-C). However, quantitative analysis showed that the compounds represented by all 11 peaks were much less abundant in cells grown in the dark (Fig. 3 D). Many additional peaks were revealed by HPLC-ESI-MS (Fig. 3 E), and MZmine resolved 218 different signals, 75 of which were putatively identified including 65 metabolites and 10 isotopes and/or fragments. The tentatively identified metabolites included 21 caffeic acid derivatives, 9 anthocyanins, 5 sucrose-derivatives, 5 sinapic acid derivatives, 4 ferulic acid derivatives, 2 coumaric acid derivatives, 13 hydroxybenzoic acid derivatives (including 3 vanillic acid derivatives), 2 flavonoids and 4 non-aromatic organic acids. The remaining 143 signals could not be identified but valuable information was nevertheless obtained because they were univocally identified by their mass to charge ratios, retention times and fragmentation patterns (MS/MS and MS^3^).

**Figure 4:**
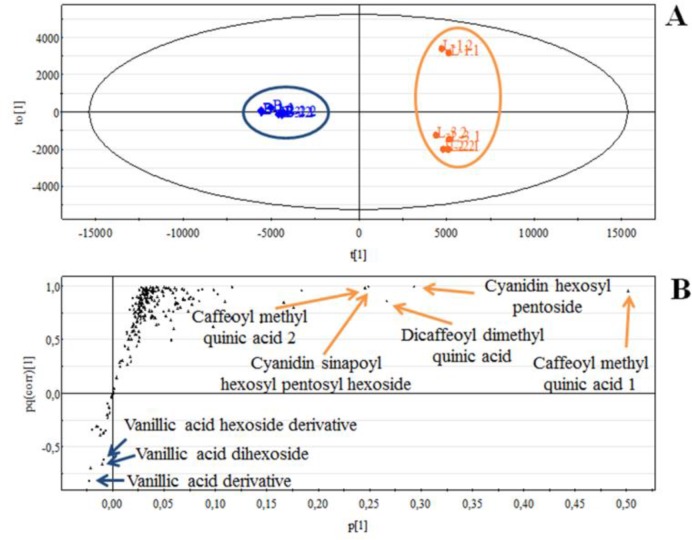
**A:** Score scatter plot of R3M carrot suspension cell cultures obtained by OPLS-DA of 218 metabolites quantified by LC-MS. Each symbol represents a single chromatography experiment, with cells grown under illumination shown in orange and those grown in the dark shown in blue. The analysis was carried out using three independent biological replicates for each treatment (i.e. L1, L2, L3 = cells cultivated in the light; B1, B2, B3 = cells cultivated in the dark) and two independent technical replicates for each biological replicate (i.e. L1.1, L1.2 are the technical replicates of biological replicate 1). **B:** S-plot of R3M carrot suspension cells cultivated in the light and in the dark, obtained by mapping *p* against *pq(corr)* in OPLS-DA. Each point represents a compound detected and quantified by LC-MS. *p* is an indicator of the “weight” of each molecule in the model, which directly depends on its abundance; *pq(corr)* indicates the ability of a compound to distinguish the dark and light samples (two classes).

Three biological and two technical replicates were analyzed for each of the two growing conditions, so the dimension of the data matrix was (3 x 2 x 2) x 218 corresponding to 2616 data points. O2PLS-DA was used to confirm the impact of light on the metabolome by imposing a two-class classification (light/dark) to generate a statistically significant model in which the light samples were clearly separated from the dark ones (Fig. 4 A). The S-plot [32] shown in Figure 4 B confirmed that light induces the accumulation of most of the secondary metabolites, especially hydroxycinnamic acids, hydroxybenzoic acids and flavonoids. Unlike HPLC-DAD, this approach also revealed molecules that were not affected by light or dark conditions, such as sugars, some hydroxycinanmic and hydroxybenzoic acids, and non-aromatic organic acids. Finally, three derivatives of vanillic acid that were not detected by HPLC-DAD were shown to accumulate specifically in cells grown in the dark. Similar experiments using cells treated under different conditions have been carried out also in basil [33] and *Echinacea angustifolia* [34].

Although the relatively simple and easily controlled environments of plant cells provide useful models, the metabolic profiles of whole plants can be modulated by their environments in a more complex manner. For example, the same spearmint chemotype (*Mentha spicata* L.) was cultivated at four sites in Turkey characterized by diverse geographical and climate conditions, resulting in a significant quantitative impact on essential oil composition. Sesquiterpenes accumulated to higher levels in plants growing in warmer areas, whereas monoterpenes accumulated to higher levels in plants growing in temperate regions [35]. Similarly, the growth of garden thyme (*Thymus vulgaris*), and the qualitative and quantitative profile of its metabolites, was shown to vary across three ecologically-diverse areas in Iran [36].

## 4) Analysis of natural remedies using LC-MS untargeted metabolomics

### 4.1) LC-MS to investigate the composition of medicinal herbs

Untargeted metabolomics has recently been used to investigate the composition of specific medicinal herbs, which can be composed by one or more herbs or parts of them. LC-DAD/ESI-MS/MS was used to determine the metabolic fingerprint of *Euonymus alatus* (Thuhb) siebold (EAS), which is recommended for the prevention of atherosclerosis. By comparing the metabolic profiles of herbs acquired at different locations, the authors found significant regional differences in the abundance of specific major and minor metabolites [37]. They also found that different tissues from the same plant (leaf, fruit, stem, and root) were qualitatively similar in terms of the metabolic profile but that individual metabolites differed in abundance, suggesting that the metabolomic analysis of different plant tissues could help to determine which is likely to have the most potent medicinal effects [37]. LC-ESI-MS/MS has also been used to analyze the rhizomes of *Dysosma versipellis* (Hance) M. Cheng, *Dysosma pleiantha* (Hance) Woodson and *Sinopodophyllum emodi* (Wall. Ex Royle) Ying [38]. These plants are commonly called *Gui-jiu* and they contain various lignans, flavonoids and steroids with health-promoting properties but also podophyllotoxin-related lignans which are highly toxic, requiring such herbal products to be carefully controlled. The authors analyzed 15 constituents in the crude extract of *D. versipellis* by LC-ESI-MS/MS in negative ion mode, and claimed that the combination of ESI-MS/MS and LC-ESI-MS/MS allowed the rapid and accurate characterization of podophyllotoxin-related glucosides and flavonoid glycosides in the crude extracts, reducing the risk of toxicity cases following the consumption of *Gui-jiu*.

### 4.2) LC-MS for the analysis of complex herbal medicines

Although untargeted LC-MS analysis has been applied to specific medicinal herbs, many traditional preparations comprise multiple herbs, so the abundance of specific bioactive compounds can vary due to the mixing ratio as well as the effect of different environments on the individual herbal components [39]. The high selectivity, sensitivity and versatility of LC-MS analysis makes it ideal also for such complex herbal medicines. For example, Chinese licorice (gān căo) is one of the oldest and most popular herbal medicines in the world, derived from the roots of *Glycyrrhiza uralensis* root and present in approximately 60% of all traditional Chinese medicine prescriptions [40, 41]. LC-ESI-MS/MS was used to identify licorice flavonoids and saponins in Si-Jun-Zi decoction, which comprises four Chinese herbs, including gān căo [42]. LC-DAD/ESI-MS was used to analyze the Chinese medicine preparation Gan-Lu-Yin, revealing 14 key compounds including liquiritigenin, liquiritin and glycyrrhizic acid from gān căo [43]. LC-MS has also been used to analyze PHY906, a modified pharmaceutical preparation of Huangqin Tang, which is used in traditional Chinese medicine to treat diarrhea, nausea and abdominal cramps [44]. PHY906 comprises four medicinal herbs – *Scutellaria baicalensis* Georgi (S), *Paeonia lactiflora Pall*. (P), *Glycyrrhiza uralensis Fisch*. (G) and *Ziziphus jujuba Mill*. (Z) – at a ratio of 3:2:2:2. LC-MS identified or tentatively characterized 64 peaks, including flavonoids, triterpene saponins and monoterpene glycosides, all of which could be assigned to the four individual herbs, and some of which were reported for the first time [45].

In the future, untargeted metabolomics could be used not only to characterize single herbs and mixtures, but also to integrate data from different experimental platforms, as explained in the concepts of *PhytomicsQC* and *Herbalome*. The sensitivity and versatility of LC-MS led to the development of the *PhytomicsQC* concept, which combines chemical analysis, bioresponse analysis and animal pharmacology to determine batch-to-batch reproducibility. This platform integrates molecular fingerprints, quantitative analysis and statistical pattern comparisons for the analysis of natural remedies [44]. The *Herbalome* project [46] aims to globalize traditional Chinese medicines by using LC-MS to determine the composition of different products and the structure and function of their components, and establishing a standard resource library to unravel the synergistic and complementary effects of these components on multiple targets.

## 5) Summary and outlook

Recently, plant-based natural remedies have become very popular in western countries, but, unlike the modern drug, their chemical composition is still partially unknown and difficult to determine. Moreover, the chemicals of herbal products can vary from batch to batch since they are largely affected by environmental growth conditions of the original plants and manufacture practices. In order to assure the safeness of such products, a deeper knowledge on these natural remedies is strongly required. LC-MS-based untargeted metabolomics is particularly suitable for the analysis of this material. The presence, amount and structures of plant metabolites can be unraveled by using this approach.

Recently, two new initiatives, *Herbalome* project and *PhytomicsQC*, have been launched to improve herbal safeness and to unify traditional Chinese medicines with western medicine by using LC-MS untargeted metabolomics for the simultaneous analysis of many compounds in herbal products.
